# Targeted drugs for systemic therapy of lung cancer with brain metastases

**DOI:** 10.18632/oncotarget.23616

**Published:** 2017-12-22

**Authors:** Ya-Wen Sun, Jian Xu, Jun Zhou, Wen-Juan Liu

**Affiliations:** ^1^ Shandong Provincial Key Laboratory of Radiation Oncology, Shandong Cancer Hospital and Institute, Shandong Cancer Hospital Affiliated to Shandong University, Shandong Academy of Medical Sciences, Jinan, China; ^2^ Shandong Cancer Hospital and Institute, Shandong Cancer Hospital Affiliated to Shandong University, Jinan, China; ^3^ Laboratory of Cancer Biology and Genetics, Center for Cancer Research, National Cancer Institute, Bethesda, MD, USA; ^4^ Institute of Medicinal Biotechnology, Chinese Academy of Medical Sciences and Peking Union Medical College, Beijing, China; ^5^ University of South Carolina, Computer Science and Engineering Department, Columbia, SC, USA

**Keywords:** brain metastases, lung cancer, target drug, EGFR-TKI, ALK

## Abstract

Brain metastases are very common in lung cancer patients. The condition of these patients is complicated and difficult to treat, and adverse reactions following treatment can affect the nervous system, which severely reduces quality of life. Lung cancers are categorized as small cell lung cancers and non-small cell lung cancers. Patients with brain metastasis of small cell lung cancers are generally treated with brain radiotherapy and systemic chemotherapy, but stage III/IV patients with brain metastasis of non-small cell lung cancers are generally not responsive to radiotherapy or chemotherapy. With the recent development of targeted drugs, tumor molecular profile detection allows the selection of appropriate targeted drugs for adjuvant pharmacological treatment of brain metastasis in lung cancer patients. In recent years, immune checkpoint inhibitors have emerged and have been approved by the Food and Drug Administration (FDA) for the treatment of certain cancers, but their efficacy in lung cancer patients with brain metastases still needs to be confirmed. This paper focuses on highlighting drugs for targeted therapy of brain metastasis in lung cancer patients and their molecular targets and mechanisms of drug resistance.

## INTRODUCTION

In lung cancer patients, metastasis of tumors to the brain is fairly common. Among all sites of lung cancer metastasis, brain metastases account for 40–50% [1, 2]. After brain metastases (BM) are detected in lung cancer patients, the median survival rate is only 3–6 months [3]. Diagnosis of brain metastases in lung cancer patients is usually based on computed tomography (CT) [4–6]. Magnetic resonance imaging (MRI) can also be used to find brain metastases at early stages in asymptomatic lung cancer patients. Patients receiving early treatment have significant improvements in quality of life.

Metastasis to the nervous system occurs in 10% of small cell lung cancer (SCLC) patients and 20–40% of non-small cell lung cancer (NSCLC) patients [7–9]. Most (80%) brain metastases occur in the cerebrum, 15% in the cerebellum, and 5% in the brain stem. The main treatment for brain metastasis in lung cancer patients is radiotherapy, which includes local brain radiotherapy and whole-brain radiation therapy (WBRT) [6, 10, 11]. However, over 35% of patients have adverse reactions to WBRT, and patients develop headaches, hemiplegia, and neurological diseases; the median overall survival (OS) is 4–11 months [7, 11–13].

Entry of cancer cells into the brain is extremely difficult, because they must pass through the blood-brain barrier (BBB) and survive in the microcirculation of the brain before growing into a tumor. Therapeutic drugs must also pass through the BBB to enter the brain of the patient [14]. The concentration of drugs in the central nervous system is an observable index that must be taken into account during drug development. Furthermore, resistance to such drugs can develop.

Treatment of lung cancer brain metastases with chemotherapeutic drugs is limited by the BBB, and toxic adverse reactions to chemotherapy also cause patients to suffer [15]. Recently, an increasing number of targeted drug therapies have been used to treat brain metastases in lung cancer patients. Kirsten ras oncogene homolog (*KRAS*) mutations, epidermal growth factor receptor (*EGFR*) mutations, and ROS proto-oncogene 1 (*ROS1*)/anaplastic lymphoma kinase (*ALK*) translocations are the four most common known genetic alterations in NSCLC. We will detail drugs designed against these molecular targets. Several small molecule targeted agents have been developed against the EGFR and ALK tyrosine kinases, which have increased hope for patients with brain metastases of lung cancer. Moreover, brain metastases in lung cancer have been shown to have relevant and sometimes divergent genetic alterations, and there has been a resurgence of interest in targeted drug delivery to the brain using standard or pulsatile dosing to achieve adequate drug concentration in the brain. Many clinical trials have demonstrated targeted drug efficacy. Using molecular targeting to treat brain metastasis has further increased the understanding of the molecular mechanisms of cancer.

### Systemic chemotherapy for small cell lung cancer

SCLC accounts for 10–15% of all lung cancers and is often accompanied by brain metastasis [8]. The usual first-line therapy for brain metastasis is platinum-based chemotherapy or WBRT [16, 17]. However, patients receiving these treatments have poor prognoses, and the chemotherapy and prophylactic cranial radiotherapy available to help prevent brain metastases are limited in efficacy. Chemotherapeutic agents for the treatment of lung cancer brain metastasis have poor efficacy, which may be due to the BBB [18]. The use of large doses leads to the exacerbation of intracranial responses, causing brain damage.

With the continued development and clinical trials of chemotherapies, the current first-line clinical treatment of brain metastasis in lung cancer patients is mainly the use of platinum-based drugs combined with etoposide or vincristine (Table 1) [19]. The intracranial response rate to carboplatin treatment alone is 40%, and its use is frequently accompanied by severe brain damage, whereas the response rate to carboplatin combined with irinotecan is 65%, with a reduction in brain damage. In addition, the response rate to etoposide combined with cisplatin (Table 1) is 37%, and the response rate to vinorelbine or gemcitabine combined with carboplatin is 45% [16, 19]. Topotecan (Table 1) is the only available second-line clinical drug for SCLCs, with an intracranial response rate of 33% [20].

**Table 1:** Chemotherapeutic regimens for brain metastasis of small cell lung cancer

Drug (trade name)	Treatment setting	Status (year approved)	Brain response rate (%)^a^	Side effects	Mechanisms of action
Cyclophosphamide	Front Line	Medical use (1959)	53%/82%^b^	Acute myeloid leukemia	Crosslinks DNA at the guanine N-7 position
Etoposide (Etopophos)	Front Line	Medical use (1983)	53%/82%^b^	Infusion site reactions, hair loss, constipation or diarrhea, metallic food taste, and bone marrow suppression	Forms complex with DNA and topoisomerase II to prevent re-ligation of the DNA strands, causes DNA strand breaks
Teniposide (Vumon)	Front Line	Phase III	22%/57%^b^	Bone marrow suppression, gastrointestinal toxicity, hypersensitivity reactions, and reversible alopecia	Inhibitor of topoisomerase II, causes DNA double-strand breaks and DNA-protein crosslinks
Doxorubicin (Adriamycin)	Front Line	Medical use (1974)	82%^b^	Cardiomyopathy, dyspigmentation, and skin eruptions	Causes double-stranded DNA breaks, blocks replication and reforming of the double helix
Vincristine (Oncovin)	Front Line	Medical use (1961)	53%/82%^b^	Peripheral neuropathy, hyponatremia, constipation, and hair loss	Binds tubulin to stop the cell from separating its chromosomes during metaphase
Cisplatin	Front Line	Medical use (1979)	85%^c^	Bone marrow suppression, hair loss, kidney toxicity, and vomiting	Interferes with DNA replication
Carboplatin (Paraplatin)	Front Line	Medical use (1986)	40%	Low blood cell levels, nausea, and electrolyte problems	Causes intra-and inter-strand DNA crosslinking
Irinotecan (Camptosar)	Front Line	Medical use (1996)	65%	Diarrhea, vomiting, bone marrow suppression, hair loss, shortness of breath, fever, and immunosuppression	Activates to SN-38, an inhibitor of topoisomerase I, inhibits DNA replication and transcription
Topotecan (Hycamtin)	Second line	FDA approved (2007)	33%	Myelosuppression, anemia, thrombocytopenia, diarrhea, nausea, vomiting, stomatitis, constipation, asthenia	Causes single-stranded DNA breaks, topoisomerase I inhibitor

^a^ Highest rate of brain response to drugs in the chemotherapy-only arm. ^b^ Brain response rate in patients administered chemotherapy plus whole-brain radiation therapy. ^c^ Brain response rate in Cisplatin followed by multidrug regimen.

### Cancer systemic chemotherapy for NSCLC

NSCLC accounts for 85–90% of all lung cancers, and brain metastasis occurs in 20–40% of patients. Unlike SCLC, stage IV NSCLC patients are not sensitive to prophylactic cranial irradiation [21], and it does not extend their median survival. In addition, the intracranial response rate of the combined use of the chemotherapy drugs carboplatin and paclitaxel is only 20% (Table 2) [22, 23]. However, during the second phase II trial, treatment of brain metastasis in NSCLC patients receiving cisplatin or pemetrexed (Table 2) combined with WBRT had brain response rates of 68.3% and 34.1%, respectively, and the median OS was 12.6 months [24]. Temozolomide is a chemotherapy drug for treating brain tumors, which can also be used to treat NSCLC brain metastasis. In general, when it is used in combination with radiotherapy, the response rate is 20% [25–27]. The efficacy of the use of this drug alone is unknown.

**Table 2:** Chemotherapeutic regimens for brain metastasis in non-small cell lung cancer

Drug (trade name)	Treatment setting	Status (year approved)	Brain response rate (%)	Side effects	Mechanisms of action
Paclitaxel (Taxol)	Front Line/Second Line	FDA approved (1993)	20%^a^	Hair loss, bone marrow suppression, numbness, allergic reactions, muscle pains, and diarrhea	Targets tubulin to suppress microtubule detachment
Pemetrexed (Alimta)	Front Line/Second Line	FDA approved (2004)	68.3%^b^	Low blood cell counts, nausea, diarrhea, oral mucositis, loss of appetite, skin rash, and constipation	Inhibits the formation of precursor purine and pyrimidine nucleotides; prevents DNA and RNA synthesis

^a.^ Brain Response Rate when using carboplatin and paclitaxel in patients with brain metastasis from non–small cell lung cancer.

^b.^ Brain Response Rate when using cisplatin and pemetrexed with concurrent whole-brain radiation therapy in patients with brain metastasis from non–small cell lung cancer.

Pemetrexed is an antifolate chemotherapy drug used to treat nonsquamous NSCLC. There are very few reports on its clinical use in treating brain metastasis in NSCLC. A study by Bearz et al. reported a partial response in 11 patients (28.2%) and stable disease in 21 (53.8%) after pemetrexed treatment in 39 patients found to have cancer cells in the central nervous system (CNS), with a clinical benefit rate of 82% for cranial metastases and an OS of 10 months [28]. Pemetrexed can cross the BBB to a limited extent. Its concentration in the cerebrospinal fluid is very low, at 0.33–1.58%. Furthermore, there are very few reports of its use to treat brain metastasis, and therefore there is a lack of clinical evidence [29].

The data above show that treatment of lung cancer patients with brain metastasis with chemotherapy drugs (Tables 1 and 2) is restricted by the limited ability of the drugs to cross the BBB, with response rates of 15–30%, a high rate of neurological adverse events, and a lack of significant improvement of median survival, usually only 4–6 months.

Recent studies have found that the ability of tumors to metastasize to other tissues and organs is related to the presence of cancer stem cells or circulating tumor cells. New chemotherapy drugs for treating lung cancer are focused on killing cancer stem cells or circulating tumor cells; they prevent the metastasis of lung cancer cells, and they can cut off the migration of circulating tumor cells to the brain after lung cancer metastasis begins and serve as an adjuvant treatment together with radiotherapy. This requires first determining the molecular profile of circulating tumor cells, using it to determine the pharmacological type of the cancer, and then selecting the appropriate chemotherapy drugs for treatment.

### Molecularly targeted therapy for brain metastasis

Use of the chemotherapy drugs discussed above is often accompanied by brain damage when used to treat brain metastasis in lung cancer patients. Radiographic findings of brain damage are also present when brain radiotherapy is used [13, 30, 31]. Repeated biopsies of brain metastases showed that the tumor tissue contains EGFR suppressors, phosphoinositide 3-kinase (PI3K)/AKT/mammalian target of rapamycin (mTOR), mitogen-activated protein kinase (MAPK), and cyclin-dependent kinase pathways, and other potential targets. Available small molecule targeted drugs fall mainly into two categories: EGFR-tyrosine kinase inhibitors (TKI) and ALK inhibitors [14, 30, 32, 33]. Treatment is selected based on determination of the molecular profile of the tumor.

### First-generation EGFR-TKIs

EGFR mutations are found in approximately 22% of all lung cancer metastases [33]. The two main signaling pathways downstream of EGFR are the RAS and PI3K pathways. The EGFR signaling pathway plays important roles in the proliferation, survival, migration, and metastasis of cancer cells. Thus, inhibition of the EGFR signaling pathway can inhibit the proliferation and metastasis of cancer cells [34, 35]. TKIs block the phosphorylation of tyrosine kinases through competitive binding, thereby inhibiting downstream signal transduction [36].

The first-generation EGFR-TKIs erlotinib (molecular weight (MW): 394 Da) and gefitinib (MW: 446 Da) have been approved by the United States Food and Drug Administration (FDA) for the treatment of metastatic NSCLC with EGFR mutations [37, 38] (Table 3). Lung cancer patients with brain metastasis who received erlotinib or gefitinib combined with radiotherapy or chemotherapy showed significantly increased intracranial response rates and significantly extended progression-free survival (PFS) and median OS compared with those who received either drug alone [39, 40]. The intracranial response rates after erlotinib or gefitinib treatment are 30–43%, and the OS is 8 months [10, 41]. In lung cancer patients with loss of *EGFR* exon 19 with brain metastasis, ^11^C labeling as a positron emission tomography marker was used to show that erlotinib can pass through the BBB and enter the brain, facilitating observation of treatment efficacy [39]. EGFR-TKIs are used to treat lung cancers with EGFR mutations, and studies have shown that EGFR-TKI treatment of patients with brain metastasis is also related to EGFR mutations [41–43]. Porta et al. evaluated 21 NSCLC patients with EGFR mutations treated with erlotinib and found that the condition of patients with brain metastasis improved without an obvious intracranial response [43]. It was recently reported that high doses of erlotinib (1500 mg/week) can control the condition of NSCLC patients with EGFR mutations and are well tolerated by patients; furthermore, no CNS metastases are observed [44, 45].

**Table 3:** EGFR TKIs for brain metastasis in non–small cell lung cancer [46]

Generation	Drug (trade name)	Treatment setting	Molecular targets	Status	EGFR inhibition	Pivotal Trials for BM	Mechanisms of drug resistance
First generation	Gefitinib (Iressa)	Second line	EGFR L858R, Del19, P-glycoprotein	Phase III (FDA approved)	Competitive; reversible	IPASS trial, Li et al. [10], Lee et al. [38]	Second EGFR mutation
	Erlotinib (Tarceva)	Second line	EGFR L858R, Del19	Phase III (FDA approved)	Competitive; reversible	BR.21, RTOG 0320, Lee et al. [38], Porta et al. [43]	EGFR T790 mutation; MET amp
second generation	Afatinib (Gilotrif)	Second line	wt-EGFR, EGFR L858R, L858R/T790M, L858R/T854A, wt-HER2, HER2 amp, HER4	Phase III (FDA approved)	Covalent; irreversible	Hoffknecht et al. [36], LUX-Lung 7/8	Second EGFR mutation (T790, L792F, C797S)
	Neratinib		EGFR L858R, T790M, EGFR (G719X), MET, Pan-ErbB, HER2, HER4	Phase III		IPASS trial (little report)	Second EGFR mutation (T790)
	Dacomitinib		EGFR L858R, Del19, T790M, wt-HER2, mutant-HER2, HER2 amp., HER4	Phase III		ARCHER 1050 trial (little report)	Second EGFR mutation (T790)
Third generation	Osimertinib (Tagrisso)	Second line	EGFR L858R, Del19, T790M (limited activity against wt-EGFR)	Phase III (FDA approved)	Covalent; irreversible	NCT02228369, BLOOM trial	Second EGFR mutation
	Rociletinib		wt-EGFR, T790M	Phase II/III (stopped)		BLOOM trial	Second EGFR mutation
	Olmutinib		wt-EGFR, EGFR L858R, Del19, T790M	Approved in South Korea^a^		HM-EMSI-101 phase I/II	Second EGFR mutation
	Nazartinib		wt-EGFR, EGFR L858R, Del19, T790M	Phase I/II		No reported	Second EGFR mutation
	Avitinib		T790M (limited activity against wt-EGFR)	Phase I		No reported	Second EGFR mutation
	AZD3759 [52]		EGFR L858R, Del19, T790M, mutant-EGFR	Phase I/II		NCT02228369	Second EGFR mutation

^a^Due to an unexpected increase of grade 3/4 skin toxicity, the ELUXA clinical trial program was temporally stopped.

Del, deletion; amp, amplification; TKIs, tyrosine kinase inhibitors; wt, wild-type; EGFR, epidermal growth factor receptor;

BM, brain metastases; MET, methyl ethyl ketone; HER/ErbB, human epidermal growth factor receptor.

### Second-generation EGFR-TKIs

Long-term treatment of lung cancer patients with EGFR mutations with first-generation EGFR-TKIs results in the development of drug resistance and decreased treatment efficacy. This is related to secondary mutations in EGFR, and the T790M mutation underlies the main mechanism of resistance [46]. Therefore, second-generation EGFR-TKIs, including afatinib, dacomitinib [47], and neratinib [48] (Table 3), were introduced. In lung cancer patients with brain metastasis treated with afatinib, there is no significant improvement in PFS compared to first-line chemotherapy drugs. The intracranial response rate for afatinib alone can reach 35% [49].

### Third-generation EGFR-TKIs

Despite the efficacy of first- and second-generation EGFR-TKIs in the treatment of BM in lung cancer patients with EGFR mutations, the disease continues to progress after a short period of time. Some benefits have been reported, but they are variable and usually do not last, probably because of the poor capability of these drugs to penetrate the BBB. Repeated biopsies show that the EGFR T790M mutation underlies the main mechanism of resistance; this mutation is present in over 50% of patients. Third-generation EGFR-TKIs include osimertinib, rociletinib, olmutinib, nazartinib, avitinib, and AZD3759 (Table 3). Osimertinib is a new EGFR-TKI targeting resistance due to the EGFR T790M mutation, and was recently approved by the FDA [50]. There have been two reports of brain metastasis in NSCLC patients with EGFR mutations treated with osimertinib, and there is a study in AURA phase I/II [51]. The two patients had already been treated with gefitinib or erlotinib and brain radiotherapy, and their condition had progressed. After osimertinib treatment, patient condition and brain damage were both controlled [52]. In 2016, results from the ASCO trial were reported [53]. Among 21 lung adenocarcinoma patients, 11 had already undergone brain radiotherapy. After daily oral administration of 160 mg osimertinib, 5 patients had significant improvement in nervous system function, and the disease was significantly controlled in 9 patients.

### ALK inhibitors

ALK rearrangements have been detected in approximately 3–7% of NSCLC cases [54]. The echinoderm microtubule-associated protein-like 4 (EML4)-ALK fusion protein was the first reported oncogenic kinase in lung cancer and is located on chromosome 2. This fusion protein includes an extracellular ligand region, a transmembrane helix region, and an intracellular domain that requires phosphorylation. After kinase activation of the protein, many oncogenes are activated by downstream signal transduction.

The first-generation ALK inhibitor crizotinib (Table 4) is taken orally [55]. It is a small molecule drug that inhibits ALK and c-Met tyrosine kinases by competing with ATP binding, thereby inhibiting the ALK-ROS1 fusion protein. In 2011, it was approved by the FDA for treatment of ALK-rearranged NSCLC patients. The PROFILE 1014 stage III, PROFILE 1005 stage II, and PROFILE 1029 stage III clinical trials all showed that, in lung cancer patients with brain metastasis, the disease control rate (DCR) of the crizotinib group was higher than the chemotherapy drug group; patients of the crizotinib group also had increased PFS and OS [55–57]. In 2010, patients taking crizotinib were found to have developed resistance [58]. Studies on the drug resistance mechanism have found that (1) ALK undergoes secondary mutations that block crizotinib from inhibiting the pathway, and (2) tumor cells can bypass ALK activation, activating this pathway by an alternate mechanism.

**Table 4:** ALK inhibitors for brain metastasis in non–small cell lung cancer

Generation	Drug (trade name)	Molecular targets	Status	Pivotal trials for BM	Mechanisms of drug resistance
First generation	Crizotinib (Xalkori)	ALK-rearranged, c-Met tyrosine kinase	Phase III (FDA approved)	PROFILE 1001, PROFILE 1005, PROFILE 1007, PROFILE 1014 phase III	Second ALK mutation (L1196M and C1156Y)
Second generation	Ceritinib (Zykadia)	ALK (L1196M, G1269A, S1206Y, F1174L, V1180L)	phase I/II (FDA approved)	ASCEND-1 phase I	Second ALK mutation (C1156Y, G1202R, F1174C, L1152R, 1151Tins, G1123S)
	Alectinib (Alecensa)	ALK (L1196M, C1156Y, G1269A, S1206Y, L1152R, F1174L, 1151Tins)	phase I/II (FDA approved)	AF-002JG phase I, NP28761 Phase II	Second ALK mutation (G1202R, V1180L, I1171T, I1171S)
Third generation	brigatinib	ALK (L1196M, C1156Y, G1202R, S1206Y, 1151Tins, D1203N, F1174C)	phase I/II	ALTA phase II	None reported
	entrectinib	ALK (L1196M, C1156Y), ROS1, NTRK1, NTRK2, NTRK-3	phase I	X-396 phase I	None reported
	lorlatinib	ALK (L1196M, G1202R, G1269A)	phase I/II	NCT01970865	None reported

ALK, anaplastic Lymphoma Kinase; TKI, tyrosine kinase inhibitor; BM: brain metastases; c-MET, cellular-mesenchymal to epithelial transition factor; NTRK, Neurotrophic Receptor Tyrosine Kinase.

Drug resistance in patients treated with crizotinib has led to the introduction of the second-generation ALK inhibitors ceritinib and alectinib for the treatment of ALK-positive NSCLC (Table 4), but adverse CNS effects for these inhibitors are greater than for the first-generation ALK inhibitors. The pharmacological mechanism of ceritinib is similar to that of crizotinib, but its ALK inhibitory activity is 20-fold that of crizotinib [59]. In 2014, the FDA approved ceritinib for the treatment of metastatic ALK-positive NSCLC patients and crizotinib-resistant patients [60]. In the ASCEND-1 phase I study of ceritinib, the brain response rate of patients not given ALK inhibitors was 42.1% and the brain DCR was 79%, whereas the brain response rate of patients given crizotinib was 18.6% and the brain DCR was 65.2% [61, 62]. The ALK inhibitory activity of alectinib is 10-fold that of crizotinib, and it can also inhibit leukocyte receptor tyrosine kinases and cyclin G-associated kinases.

In 2015, the FDA approved alectinib (Table 4) for the treatment of metastatic ALK-positive NSCLC patients and crizotinib-resistant patients [63]. In the phase I/II AF-001JP clinical trial, the maximum tolerated dose was 300 mg, the brain DCR of alectinib-treated lung cancer patients with brain metastasis was 90%, the brain response rate was 52.4%, and the complete response rate was 29% [63–65]. Currently, alectinib treatment of brain metastasis in lung cancer patients has very good clinical efficacy. The phase II NP28761 clinical trial reported that intracranial DCR was 100%.

At the 2016 ASCO annual meeting, brigatinib (Table 4) was reported to be developed for crizotinib-resistant ALK-positive NSCLC patients [66]. The activity of brigatinib in the treatment of brain metastasis in cancer is relatively good. Its targets are EGFR, ALK, ROS1, and EGFR T790M. A total of 222 NSCLC patients, of whom 154 had brain metastasis and 164 underwent chemotherapy, were randomly divided 1:1 into two groups: group A received 90 mg brigatinib daily, and group B received 90 mg for 7 days followed by 180 mg daily. The objective response rate (ORR) of group B was 67%, whereas the ORR of group A was only 42%. The average PFS was 9.2 months for group A and 12.9 months for group B [67, 68].

The third-generation ALK inhibitors lorlatinib and entrectinib are still in clinical trials [69]. Entrectinib (X-396) is used to treat ALK-positive NSCLC patients, and its targets are ALK, ROS1, NTRK1 (Neurotrophic Receptor Tyrosine Kinase 1), NTRK2, and NTRK-3. Lorlatinib has been reported to be effective in lung cancer patients with brain metastasis, with an ORR in target lesions reaching 60% in ALK/ROS1 translocated tumors [70, 71].

### Other targetable molecular alterations

*ROS1* fusion genes were found in 1–2% of lung adenocarcinoma patients [72]. ROS1-positive lung adenocarcinoma patients have been treated with a combination of crizotinib and lorlatinib (Table 5), but the data have not yet been published [73–75]. The BRAF inhibitor dabrafenib (Table 5) and the MEK (methyl ethyl keytone) inhibitor trametinib have been used clinically to treat BRAF-positive lung adenocarcinoma patients [76–78]. Dabrafenib and vemurafenib [79, 80] have been approved by the FDA for the treatment of melanoma with nervous system metastasis. There have been clinical reports of dabrafenib being used to treat lung cancer patients with brain metastasis, but further clinical validation is needed.

**Table 5:** Other molecular targeted therapies for brain metastasis in non-small lung cancer

Drug (trade names)	Molecular targets	Targeted mutated sites in NSCLC	Status	Treatment strategies for BM in NSCLC	Mechanisms of drug resistance
Dabrafenib (Tafinlar)	в-Raf	BRAF V600E/K-mutant	FDA approved	Combination with trametinib (BRAF V600-positive)	Overexpression of PDGFRB, NRAS mutation
Vemurafenib (Zelboraf)	в-Raf	BRAF V600E/K-mutant	FDA approved	Combination of dabrafenib and trametinib	Overexpression of PDGFRB, second NRAS mutation
Cabozantinib (Cabometyx)	c-Met, VEGFR-2, AXL, RET	RET fusion-positive	FDA granted orphan drug status	Combination with everolimus	None reported
Bevacizumab (Avastin)	VEGF-A	VEGF positive	FDA approved	Combination with carboplatin/paclitaxel	VEGF-D, VEGF-C
Ramucirumab (Cyramza)	VEGFR-2	VEGFR positive	FDA approved	Combination with carboplatin/paclitaxel	VEGFR-2 mutant
Nivolumab (Opdivo)	PD-1	BRAF mutant	FDA approved	Combination with ipilimumab	No reports
Pembrolizumab (Keytruda)	PD-1	PD-L1 overexpression, no mutations in EGFR or in ALK	FDA approved	Pembrolizumab	No reports
Atezolizumab (Tecentriq)	PD-L1	Cytotoxic T-cells	FDA approved	Atezolizumab	No reports
Ipilimumab (Yervoy)	CTLA-4	Cytotoxic T lymphocytes	FDA approved	Combination with carboplatin	No reports

NSCLC, non-small cell lung cancer; BM, brain metastases; VEGFR-2, vascular endothelial growth factor receptor 2; VEGF: vascular endothelial growth factor; PD-1, programmed cell death protein 1; PD-L1, Programmed death-ligand 1; EGFR: epidermal growth factor receptor; ALK, anaplastic lymphoma kinase; CTLA-4, cytotoxic T-lymphocyte-associated protein 4.

### Angiogenesis inhibitors

**Figure 1:**
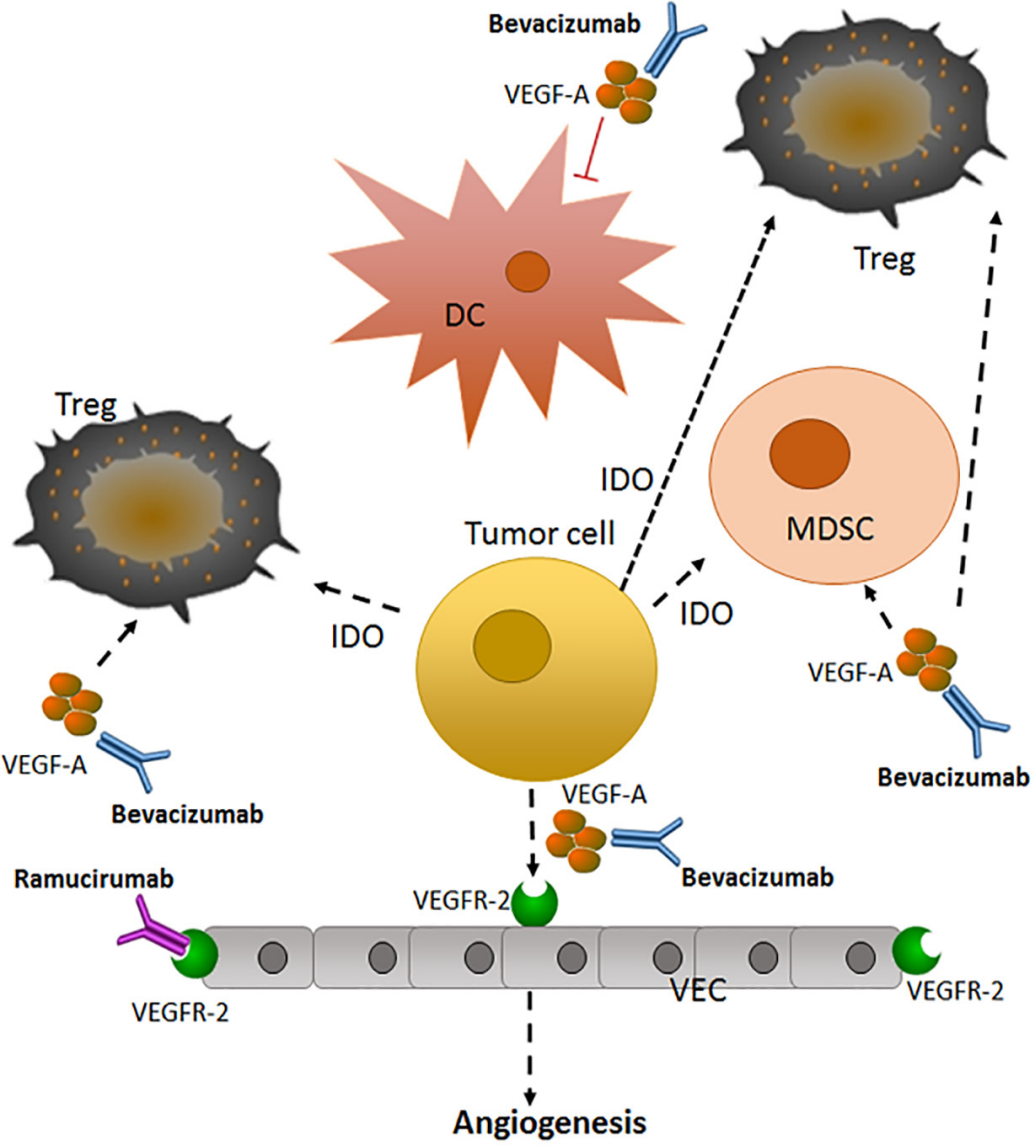
Anti-angiogenic therapy in advanced or metastatic NSCLC [81] Tumor cells can secrete VEGF to promote angiogenesis, a necessary step for tumor growth and metastasis. This secreted VEGF can activate VEGFR-2 on endothelial cells, promoting the growth of new blood vessels, as well as activating signaling pathways in immune cells. Bevacizumab and ramucirumab target VEGF-A and VEGFR-2, respectively, to prevent angiogenesis. VEGF-A, vascular endothelial growth factor A; Treg, T- regulatory cell; DC: dendritic cell; VEC: vascular endothelial cell; IDO, indoleamine 2, 3 -dioxygenase; MDSC, myeloid-derived suppressor cell; VEGFR-2: vascular endothelial growth factor receptor-2.

Tumor angiogenesis promotes NSCLC invasion and metastasis, and recently has been considered a target for treatment [81] (Figure 1). Vascular endothelial growth factor (VEGF) and other vascular growth factors, including fibroblast growth factor and platelet-derived growth factor, initiate the formation of new blood vessels and change the tumor microenvironment [82, 83]. Currently, the anti-angiogenesis monoclonal antibodies bevacizumab and ramucirumab, targeting VEGF-A and its receptor (VEGFR-2), respectively, (Table 5) have been approved for the treatment of nonsquamous metastatic NSCLC [84, 85].

### Bevacizumab

The PASSPORT trial primarily evaluated bevacizumab treatment of nonsquamous NSCLC patients, including those with brain metastasis [86–90]. Bevacizumab treatment was administered in combination with the chemotherapy drugs paclitaxel-carboplatin or erlotinib [87, 91]. Among 39 patients undergoing treatment, brain metastasis was found in 11 patients. After 6 weeks of treatment with bevacizumab and chemotherapy, the median PFS was 8.2 months and the OS was 14 months, which was not significantly different from patients without brain metastasis. Furthermore, there were no cases of contingencies or CNS hemorrhage.

The NCT02959749 is a phase III clinical trial [88]; among 147 NSCLC patients with confirmed non-squamous lung cancer, metastasis, and EGFR T790M mutations, 74 were given osimertinib (80 mg/day) and 73 were given docetaxel (75 mg/m^2^) and bevacizumab (7.5 mg/kg) treatment. Patients were provided treatment until their condition progressed or unexpected toxic reactions occurred. The PFS was 10.2 months in the osimertinib group and 2.95 months in the docetaxel-bevacizumab group. At the 2017 ASCO conference, many clinical trials of bevacizumab treatment of EGFR-TKI-resistant NSCLC with EGFR mutations and metastasis were reported. The combination of afatinib and bevacizumab was effective, suggesting a new regimen for the treatment of EGFR-TKI-resistant NSCLC patients, but more clinical data are required.

### Ramucirumab

The REVEL trial evaluated the efficacy of ramucirumab and chemotherapy drugs used as second-line treatment in metastatic NSCLC [92–94]. The evaluation showed that ramucirumab combined with docetaxel was the most effective. In a randomized phase II trial of Japanese NSCLC patients, the PFS was 5.2 months compared with 4.2 months for placebo plus docetaxel, and the OS was 15.5 months compared with 14.7 months for placebo plus docetaxel. Based on these data, ramucirumab and docetaxel were approved by the FDA for second-line treatment of metastatic NSCLC.

### Immune checkpoint inhibitors

Immune checkpoints are dysregulated in malignancies such as lung cancer. Cancer patients with brain metastasis have high rates of tumor lymphocyte invasion in the brain microenvironment [95]. However, the use of immune checkpoint inhibitors in NSCLC patients with brain metastasis is limited [96].

Nivolumab [97], pembrolizumab [98], and atezolizumab [99] with docetaxel have been approved by the FDA as second-line therapies for the treatment of NSCLC that has failed treatment with platinum-based chemotherapy drugs (Table 5). Pembrolizumab is an anti-PD-1 antibody evaluated in the NCT02085070 phase II clinical trial. A dose of 10 mg/kg pembrolizumab was given to 10 NSCLC patients with brain metastasis for 2 weeks, which was effective in 9 patients, of which 4 had particularly good reactions; 1 patient experienced brain damage. A total of 18 NSCLC patients (PD-L1-positive) received continued pembrolizumab treatment. The safety of 4 weeks of continued use was evaluated, and brain MRI was used to evaluate reactions on the 8^th^ week. The brain response rate of 6 patients with brain metastasis was 33%.

There are few clinical studies on the anti-PD-L1 antibody nivolumab. At the 2016 ESMO conference, it was reported that, among 372 squamous NSCLC patients, 38 patients had brain metastasis, and the DCR was 47% among all patients and 47.3% among patients with brain metastasis. Furthermore, the average PFS and OS of patients with brain metastasis were 5.5 months and 6.5 months, respectively. In a randomized phase III clinical trial, the anti-PD-L1 antibody atezolizumab combined with docetaxel was effective in controlling disease progression in NSCLC patients compared to chemotherapy alone. In addition, it extended the overall survival of patients, and no patients died during the course of administration.

The anti-CTLA-4 antibody ipilimumab [100, 101] increases the number of cytotoxic T cells, and is very effective in treating brain metastasis of melanoma (Table 5). Recently, there have been reports of treatment of brain metastasis in lung cancer using these antibodies, mostly with the combination of pembrolizumab and nivolumab.

## CONCLUSIONS

Brain radiotherapy is the first treatment for brain metastasis in SCLC patients. If control cannot be achieved and the disease progresses, adjuvant treatment with platinum-based first-line chemotherapy drugs or chemotherapy alone should be used. In NSCLC patients with brain metastasis, chemotherapy cannot substitute for radiotherapy, because patients with brain metastasis are not sensitive to chemotherapy. In the current era of treatment of NSCLC with targeted drugs and immune checkpoint inhibitors, if patients meet criteria such as EGFR-mutated or ALK-positive NSCLC brain metastasis, the combination of tyrosine kinase small molecule inhibitors and radiotherapy or chemotherapy should be considered a priority. The efficacy of immunotherapy of brain metastasis in NSCLC patients has not yet been confirmed and requires further support with clinical data. Currently, the emergence of liquid biopsy and circulating tumor cell detection has permitted early diagnosis and the possibility of early treatment of brain metastases in lung cancer patients. However, the available targeted drugs for the treatment of brain metastasis in lung cancer patients benefit only some of these patients and are expensive, and further studies on the molecular mechanisms of cancer are needed to find additional targets.
